# Supplementary material for: The Complex Dynamic of Phase I Drug Metabolism in the Early Stages of Doxorubicin Resistance in Breast Cancer Cells
Source: Genes (Basel). 2022 Oct 29;13(11):1977. doi: 10.3390/genes13111977 (PMC9689592; doi:10.3390/genes13111977)
Supplement: Supplementary file 1 [file genes-13-01977-s001.zip › genes-1966357-supplementary.pdf]

Supplementary Material

The complex dynamic of Phase I Drug Metabolism in the early stages of doxorubicin resistance in breast cancer cells.

Isabel S. Barata [1], Bruno C. Gomes [1], António S. Rodrigues [1], José Rueff [1], Michel Kranendonk [1], Francisco Esteves [1,*].

[1] Center for Toxicogenomics & Human Health (ToxOmics), NOVA Medical School/Faculty of Medical Sciences, Universidade NOVA de Lisboa, Lisboa, Portugal.

* Correspondence: francisco.esteves@nms.unl.pt

Supplementary Tables

Table S1. List of targets assayed by the TaqMan™ Array Human CYP450 & other Oxygenases.

GENE SYMBOL	GENE NAME
**18S**	Eukaryotic 18S rRNA
**GAPDH**	glyceraldehyde-3-phosphate dehydrogenase
**HPRT1**	hypoxanthine phosphoribosyltransferase 1 (Lesch-Nyhan syndrome)
**GUSB**	glucuronidase, beta
**CYP1A1**	cytochrome P450, family 1, subfamily A, polypeptide 1
**CYP1A2**	cytochrome P450, family 1, subfamily A, polypeptide 2
**CYP1B1**	cytochrome P450, family 1, subfamily B, polypeptide 1
**CYP11A1**	cytochrome P450, family 11, subfamily A, polypeptide 1
**CYP11B1**	cytochrome P450, family 11, subfamily B, polypeptide 1
**CYP11B2**	cytochrome P450, family 11, subfamily B, polypeptide 2
**CYP17A1**	cytochrome P450, family 17, subfamily A, polypeptide 1
**CYP19A1**	cytochrome P450, family 19, subfamily A, polypeptide 1
**CYP2A13**	cytochrome P450, family 2, subfamily A, polypeptide 13
**CYP2A6**	cytochrome P450, family 2, subfamily A, polypeptide 6
**CYP2A7**	cytochrome P450, family 2, subfamily A, polypeptide 7
**CYP2B6**	cytochrome P450, family 2, subfamily B, polypeptide 6
**CYP2C18**	cytochrome P450, family 2, subfamily C, polypeptide 18
**CYP2C19**	cytochrome P450, family 2, subfamily C, polypeptide 19
**CYP2C8**	cytochrome P450, family 2, subfamily C, polypeptide 8
**CYP2C9**	cytochrome P450, family 2, subfamily C, polypeptide 9
**CYP2D6**	cytochrome P450, family 2, subfamily D, polypeptide 6
**CYP2E1**	cytochrome P450, family 2, subfamily E, polypeptide 1
**CYP2F1**	cytochrome P450, family 2, subfamily F, polypeptide 1
**CYP2J2**	cytochrome P450, family 2, subfamily J, polypeptide 2
**CYP2R1**	cytochrome P450, family 2, subfamily R, polypeptide 1
**CYP2S1**	cytochrome P450, family 2, subfamily S, polypeptide 1
**CYP2U1**	cytochrome P450, family 2, subfamily U, polypeptide 1
**CYP2W1**	cytochrome P450, family 2, subfamily W, polypeptide 1
**CYP3A4**	cytochrome P450, family 3, subfamily A, polypeptide 4
**CYP3A43**	cytochrome P450, family 3, subfamily A, polypeptide 43
**CYP3A5**	cytochrome P450, family 3, subfamily A, polypeptide 5
**CYP3A7**	cytochrome P450, family 3, subfamily A, polypeptide 7
**CYP4A11, CYP4A22**	cytochrome P450, family 4, subfamily A, polypeptide 11, cytochrome P450, family 4, subfamily A, polypeptide 22
**CYP4B1**	cytochrome P450, family 4, subfamily B, polypeptide 1
**CYP4F11**	cytochrome P450, family 4, subfamily F, polypeptide 11
**CYP4F12**	cytochrome P450, family 4, subfamily F, polypeptide 12
**CYP4F2**	cytochrome P450, family 4, subfamily F, polypeptide 2
**CYP4F22**	cytochrome P450, family 4, subfamily F, polypeptide 22
**CYP4F3**	cytochrome P450, family 4, subfamily F, polypeptide 3
**CYP4F8**	cytochrome P450, family 4, subfamily F, polypeptide 8
**CYP4V2**	cytochrome P450, family 4, subfamily V, polypeptide 2
**CYP4X1**	cytochrome P450, family 4, subfamily X, polypeptide 1
**CYP4Z1**	cytochrome P450, family 4, subfamily Z, polypeptide 1
**CYP7A1**	cytochrome P450, family 7, subfamily A, polypeptide 1
**CYP7B1**	cytochrome P450, family 7, subfamily B, polypeptide 1
**CYP8B1**	cytochrome P450, family 8, subfamily B, polypeptide 1
**CYP20A1**	cytochrome P450, family 20, subfamily A, polypeptide 1

**CYP21A2**	cytochrome P450, family 21, subfamily A, polypeptide 2
**CYP24A1**	cytochrome P450, family 24, subfamily A, polypeptide 1
**CYP26A1**	cytochrome P450, family 26, subfamily A, polypeptide 1
**CYP26B1**	cytochrome P450, family 26, subfamily B, polypeptide 1
**CYP26C1**	cytochrome P450, family 26, subfamily C, polypeptide 1
**CYP27A1**	cytochrome P450, family 27, subfamily A, polypeptide 1
**CYP27B1**	cytochrome P450, family 27, subfamily B, polypeptide 1
**CYP27C1**	cytochrome P450, family 27, subfamily C, polypeptide 1
**CYP39A1**	cytochrome P450, family 39, subfamily A, polypeptide 1
**CYP46A1**	cytochrome P450, family 46, subfamily A, polypeptide 1
**CYP51A1**	cytochrome P450, family 51, subfamily A, polypeptide 1
**TBXAS1**	thromboxane A synthase 1 (platelet, cytochrome P450, family 5, subfamily A)
**PTGIS**	prostaglandin I2 (prostacyclin) synthase
**POR**	P450 (cytochrome) oxidoreductase
**CYB5A**	cytochrome b_5 type A (microsomal)
**CYB5B**	cytochrome b5 type B (outer mitochondrial membrane)
**CYB5R1**	cytochrome b_5 reductase 1
**CYB5R2**	cytochrome b_5 reductase 2
**CYB5R3**	cytochrome b_5 reductase 3
**CYB5R4**	cytochrome b_5 reductase 4
**FDX1**	ferredoxin 1
**FDXR**	ferredoxin reductase
**FLAD1**	FAD1 flavin adenine dinucleotide synthetase homolog (*S. cerevisiae*)
**NENF**	neuron derived neurotrophic factor
**PGRMC1**	progesterone receptor membrane component 1
**PGRMC2**	progesterone receptor membrane component 2
**BCMO1**	beta-carotene 15,15'-monooxygenase 1
**DBH**	dopamine beta-hydroxylase (dopamine beta-monooxygenase)
**DOHH**	deoxyhypusine hydroxylase/monooxygenase
**FMO1**	flavin containing monooxygenase 1
**FMO2**	flavin containing monooxygenase 2 (non-functional)
**FMO3**	flavin containing monooxygenase 3
**FMO4**	flavin containing monooxygenase 4
**FMO5**	flavin containing monooxygenase 5
**KMO**	kynurenine 3-monooxygenase (kynurenine 3-hydroxylase)
**PAH**	phenylalanine hydroxylase
**PAM**	peptidylglycine alpha-amidating monooxygenase
**SC4MOL**	sterol-C4-methyl oxidase-like
**SQLE**	squalene epoxidase
**TH**	tyrosine hydroxylase
**TPH1**	tryptophan hydroxylase 1 (tryptophan 5-monooxygenase)
**TPH2**	tryptophan hydroxylase 2
**TYR**	tyrosinase (oculocutaneous albinism IA)
**MAOA**	monoamine oxidase A
**MAOB**	monoamine oxidase B
**ALOXE3**	arachidonate lipoxygenase 3
**ALOX5**	arachidonate 5-lipoxygenase
**ALOX12**	arachidonate 12-lipoxygenase
**ALOX15**	arachidonate 15-lipoxygenase
